# A New Immunosuppressive Molecule Emodin Induces both CD4^+^FoxP3^+^ and CD8^+^CD122^+^ Regulatory T Cells and Suppresses Murine Allograft Rejection

**DOI:** 10.3389/fimmu.2017.01519

**Published:** 2017-11-08

**Authors:** Feifei Qiu, Huazhen Liu, Chun-Ling Liang, Golay D. Nie, Zhenhua Dai

**Affiliations:** ^1^Section of Immunology, Guangdong Provincial Academy of Chinese Medical Sciences, Guangdong Provincial Hospital of Chinese Medicine, Guangzhou, China; ^2^School of Medicine, University of Texas Medical Branch, Galveston, TX, United States

**Keywords:** transplant immunology, regulatory T cell, T cell, immunosuppression, allograft rejection

## Abstract

Due to vigorous alloimmunity, an allograft is usually rejected without any conventional immunosuppressive treatment. However, continuous global immunosuppression may cause severe side effects, including tumors and infections. Mounting evidence has shown that cyclosporine (CsA), a common immunosuppressant used in clinic, impedes allograft tolerance by dampening regulatory T cells (Tregs), although it inhibits allograft rejection at the same time. Therefore, it is necessary to seek an alternative immunosuppressive drug that spares Tregs with high efficiency in suppression but low toxicity. In this study, we investigated the capacity of emodin, an anthraquinone molecule originally extracted from certain natural plants, to prolong transplant survival in a mouse model and explored the cellular and molecular mechanisms underlying its action. We found that emodin significantly extended skin allograft survival and hindered CD3^+^ T cell infiltration in the allograft, accompanied by an increase in CD4^+^Foxp3^+^ and CD8^+^CD122^+^ Treg frequencies and numbers but a reduction in effector CD8^+^CD44^high^CD62L^low^ T cells in recipient mice. Emodin also inhibited effector CD8^+^ T cells proliferation *in vivo*. However, CD4^+^CD25^+^, but not CD8^+^CD122^+^, Tregs derived from emodin-treated recipients were more potent in suppression of allograft rejection than those isolated from control recipients, suggesting that emodin also enhances the suppressive function of CD4^+^CD25^+^ Tregs. Interestingly, depleting CD25^+^ Tregs largely reversed skin allograft survival prolonged by emodin while depleting CD122^+^ Tregs only partially abrogated the same allograft survival. Furthermore, we found that emodin hindered dendritic cell (DC) maturation and reduced alloantibody production posttransplantation. Finally, we demonstrated that emodin inhibited *in vitro* proliferation of T cells and blocked their mTOR signaling as well. Therefore, emodin may be a novel mTOR inhibitor that suppresses alloimmunity by inducing both CD4^+^FoxP3^+^ and CD8^+^CD122^+^ Tregs, suppressing alloantibody production, and hindering DC maturation. Thus, emodin is a newly emerging immunosuppressant and could be utilized in clinical transplantation in the future.

## Introduction

Organ transplantation is an essential and efficient approach to replacing a dysfunctional organ in patients suffering from an end-stage organ disease. However, allogeneic transplantation induces a series of allograft rejection episodes and immune responses, which are mainly mediated by alloreactive T cells (1, 2). Therefore, global immunosuppressive agents, such as cyclosporine (CsA), are needed to stop acute allograft rejection by suppressing T cell activation. On the other hand, regulatory T cells (Tregs) play a critical role in maintaining allograft survival or tolerance by inhibiting alloreactive T cell activation (3) while a reduction in their generation or activation contributes to allograft rejection (4). Although conventional immunosuppressants can prevent acute rejection, they may also cause severe side effects, including tumors and infections. Furthermore, a global immunosuppressive agent, such as CsA, inhibits the generation and function of Tregs (5, 6), likely hindering tolerance induction. Although another global immunosuppressant rapamycin, a typical mTOR inhibitor, can spare Tregs (7, 8), it may still cause same side effects as other global immunosuppressive agents do. Therefore, it is imperative to seek an alternative immunosuppressant that does not compromise Tregs, yet with high efficiency in suppression, low toxicity, and high affordability.

Emodin, 1,3,8-trihydroxy-6-methylanthraquinone, is an active anthraquinone originally isolated from certain natural plants, including *Rheum palmatum* (9) and *Cassia obtusifolia* (10). Emodin has long been used to treat chronic kidney diseases with few side effects (11). Recently, many studies have also documented laxative effects of emodin as well as its inhibitory effects on tumors (12), viruses (13), bacteria (14), and inflammation (15) without any major side effect. Furthermore, emodin is also available in China at a low cost. Therefore, emodin may exhibit unique advantages over other mTOR inhibitors in terms of the side effect issue and affordability.

Two previous publications have demonstrated that emodin inhibits acute allograft rejection after liver transplantation in an animal model (16, 17). Nevertheless, the cellular and molecular mechanisms underlying its suppression of liver allograft rejection are unknown. It is also unclear whether emodin suppresses rejection of other types of organ transplants beyond a liver allograft, which may develop spontaneous tolerance. In this study, we found that emodin significantly prolonged survival of a skin allograft, a stringent transplant model. Combined treatments with both emodin and CsA further extended the skin allograft survival. Emodin significantly increased the frequencies of both CD4^+^CD25^+^ and CD8^+^CD122^+^ Tregs while reducing the numbers of effector CD8^+^ T cells. Furthermore, CD4^+^CD25^+^ Tregs isolated from emodin-treated recipient mice were more potent in suppression of allograft rejection than those derived from control recipients, suggesting that emodin enhances CD4^+^CD25^+^ Treg function. Emodin also suppressed alloantibody production and hindered dendritic cell (DC) maturation posttransplantation. Finally, we demonstrated that emodin inhibited T cell proliferation *in vitro* and blocked their mTOR signal transduction.

## Materials and Methods

### Animals

BALB/c and C57BL/6 male mice (6–8 weeks old, male) were obtained from Guangdong Medical laboratory Animal Center (Guangdong, China) while Rag1^−/−^ mice (B6 background) were purchased from Jackson Laboratory (Bar Harbor, ME, USA). All mice were housed under a specific pathogen-free condition. All experiments were performed according to the Chinese national guidelines for the Care and Use of Laboratory Animals and approved by the Institutional Animal Care and Use Committee of Guangdong Provincial Academy of Chinese Medical Sciences (Ref. No: 201).

### Skin Transplantation

Skin donors were 7- to 8-week-old wild-type BALB/c mice (male), while skin graft recipients were 7- to 8-week-old-male C57BL/6 mice. Round-shaped full-thickness trunk skin with an approximate size of 10 mm^2^ was transplanted to the dorsal flank area of a recipient mouse and secured with a bandage of Band-Aid (Johnson Johnson, New Brunswick, NJ, USA). The bandage was removed 8 days after transplantation. Skin allograft rejection was monitored daily and defined as graft necrosis greater than 90%, as described in our previous publication (18).

### Treatment of Mice

Mice were randomly divided into control groups and groups treated with emodin (10 mg/kg body weight), cyclosporine (CsA: 20 mg/kg body weight), and emodin plus CsA for four consecutive weeks or until graft rejection/sample collection. CsA and emodin (Sigma) were prepared with saline and sodium carboxymethyl cellulose (CMC-Na; Sigma), respectively. Control groups received CMC-Na orally. Our primary data showed that oral administration of CMC-Na did not alter allograft rejection compared to untreated groups (unpublished observation). Emodin was also administered orally while CsA was given through intraperitoneal injection. To deplete Tregs, recipient mice were treated i.p. with anti-CD25 Ab (Clone: PC61, eBioscience) at 0.2 mg on days 0, 3, 6, and 10 or anti-CD122 Ab (Clone: TMβ1, eBioscience) at 0.1 mg on days 0, 7, and 14 posttransplantation as described previously (19, 20).

### Immunohistochemistry

Allografts were fixed with 4% paraformaldehyde for 24 h and then embedded in paraffin. Tissues were cut into 3-μm thick sections and placed on slides. The sections were then used for H&E and immunohistochemistry staining. For immunohistochemistry, slides with sections were incubated with primary anti-CD3 antibody (1:100, Abcam) at 4°C overnight, then secondary antibody HRP-anti-Rabbit IgG (Maxim), followed by diaminobenzidine color development. For quantitative analyses, slides were imaged at a magnification of 200×. The area of inflammation/infiltration and integrated optical density (IOD) of CD3 were measured using ImagePro plus 6 software.

### Flow Cytometry

Draining lymph node (LN) and spleen cells were harvested and stained with anti-CD4-FITC (Clone H129.19)/CD8-FITC (Clone 53-6.7), CD11c-PE (Clone HL3), CD44-V450 (Clone IM7), CD62L-APC (Clone MEL-14), CD80-FITC (Clone 16-10A1), CD86-FITC (Clone GL1), and anti-CD122-PE antibodies (Clone TM-Beta 1) (all from BD Biosciences). To determine intracellular FoxP3 expression, cells were fixed and permeated according to the protocol of Foxp3/Transcription Factor Fixation/Permeabilization Concentrate and Diluent Kit (eBioscience). Then, cells were stained with anti-FoxP3-APC antibody (Clone FJK-16s, eBioscience) and finally analyzed by a flow cytometer (FACSCalibur, BD Biosciences). To purify CD4^+^CD25^+^ and CD8^+^CD122^+^ Tregs for adoptive transfer experiments, spleen cells were stained with anti-CD4-PE (Clone RM4-5) and anti-CD25-FITC (Clone 3C7) or, separately, anti-CD8-PE (Clone 53-6.7) and anti-CD122-FITC (Clone TM-Beta 1) Abs (BD Biosciences). CD4^+^CD25^+^ or CD8^+^CD122^+^ cells were then sorted out by FACSAria III (BD Biosciences). The purity of the sorted cells was typically >95%.

### *In Vivo* Analysis of T Cell Proliferation and Apoptosis

Recipient mice were pulsed intraperitoneally with 0.1 mg of 5-ethynyl-2′-deoxyuridine (EDU, RIBOBIO) in PBS 10 days after transplantation. 24 h later, spleen and LN cells were isolated and single-cell suspensions were prepared for EDU detection using Cell-Light™ Apollo^®^488 Stain Kit (RIBOBIO) according to the manufacturer’s instructions. Then, cells were stained for cell surface markers with anti-CD8-APC-Cy7 (Clone 53-6.7), CD44-V450 (Clone IM7), and CD62L-APC Abs (Clone MEL-14) (all from BD Biosciences). To detect cell apoptosis, cells were stained for the same surface markers and then Annexin V-FITC according to the protocol of Annexin V-FITC Apoptosis Detection Kit (BD Pharmingen). Cells finally were analyzed *via* a flow cytometer (FACSCalibur).

### Analysis of T Cell Proliferation *In Vitro*

FACS-sorted CD3^+^ T cells (2 × 10^5^/well), derived from B6 mice, were cultured with irradiated Balb/c spleen cells (2 × 10^5^/well) in the absence or presence CsA (2.5 µg/ml) or emodin (50 µM) in 96-well plates (Corning Costar) in complete RPMI 1640 medium (10%FCS, 2 mM glutamine, 100 U/ml penicillin, and 100 µg/ml streptomycin) for 3 and 5 days, respectively. Cells were pulsed with [^3^H]-thymidine at 0.5 μCi per well for last 8 h. They were finally harvested and analyzed by a Scintillation counter (Perkin Elmer, Wellesley, MA, USA).

### Alloantibody Assay

Spleen cells derived from BALB/c mice were harvested and their red blood cells were lysed. B cells in splenocytes were first depleted using B220 microbeads (Miltenyi Biotec) *via* negative selection. Cells (1 × 10^6^/sample) then were stained with diluted serum (1/10) from naïve or transplanted C57BL/6 mice. They were further incubated with PE-anti-mouse IgM or FITC-anti-mouse IgG (Biolegend). The mean fluorescence intensity was determined by a flow cytometer (FACSCalibur, BD Biosciences).

### Western Blotting

Cultured T cells were lysed in RIPA buffer (50 mM Tris pH 7.5, 150 mM NaCl, 1% Triton X-100, and 5 mM ethylenediaminetetraacetic acid). Cell protein was extracted using RIPA buffer and protein concentration was measured using BCA Kit (Pierce, IL, USA). Samples were run on 10% SDS-PAGE gels and transferred onto a PVDF membrane. TBST with 5% milk was used to block the membrane, which was then incubated with a primary antibody anti-P70S6K or anti-phospho-P70S6K (1:1,000; Cell Signaling Technology) at 4°C overnight. After the incubation, membranes were washed and incubated with a secondary antibody, HRP-conjugated anti-rabbit IgG (1:10,000, Abbkine), for 1 h. GAPDH (1:1,000, Cell Signaling Technology) was also used for loading controls. Finally, signals were detected by an ECL method (Promega) and analyzed by Image J Program software.

### Statistical Analyses

Comparisons of the means were performed using Student’s *t*-test and one-way ANOVA. Data were presented as the mean ± SD and analyzed through GraphPad Prism 6 (GraphPad Software, La Jolla, CA, USA). The analysis of graft survival was conducted using Kaplan–Meier method (log-rank test). A value of *P* < 0.05 was considered statistically significant.

## Results

### Emodin Significantly Prolongs Skin Allograft Survival

**Figure 1:**
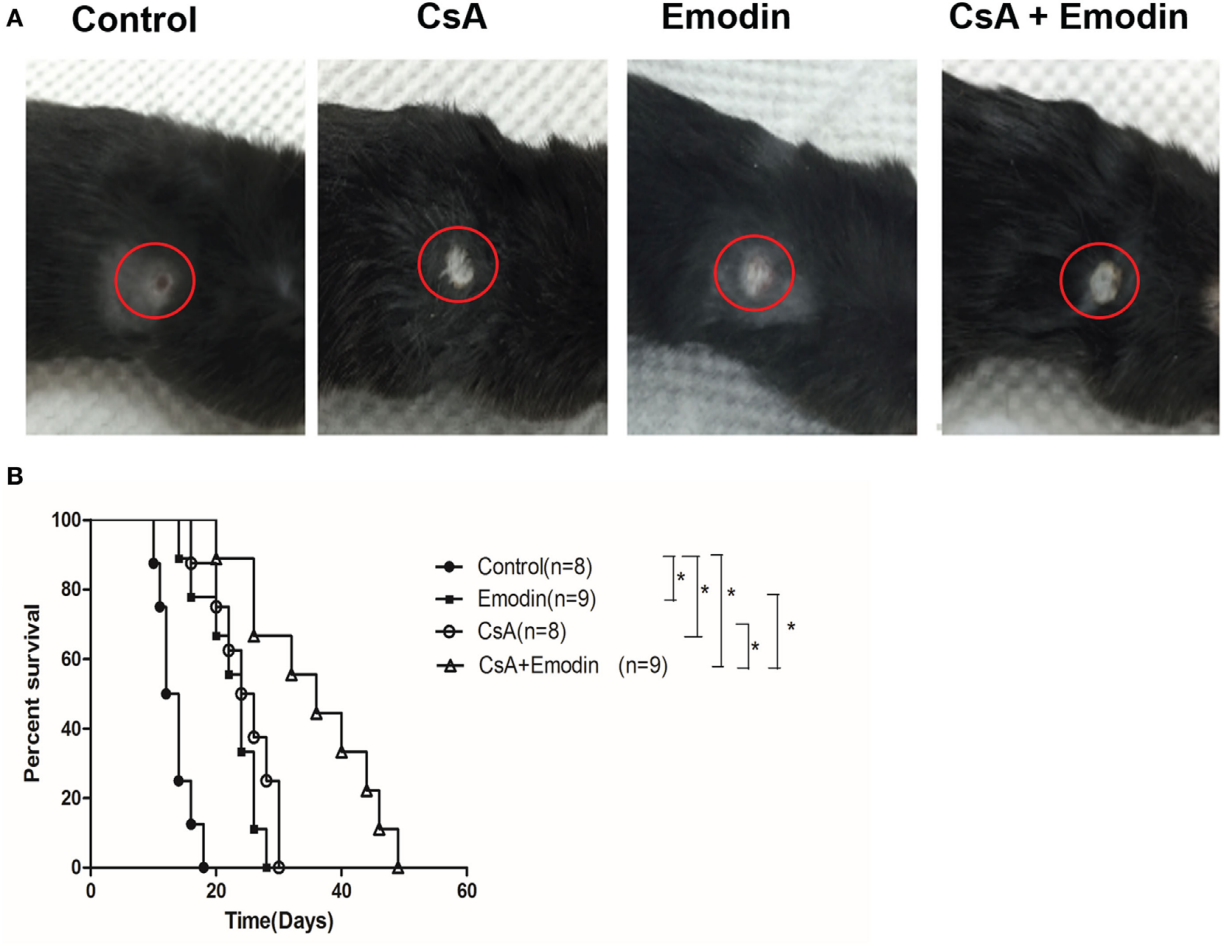
Emodin prolongs skin allograft survival. **(A)** Representatives of BALB/c-derived skin allografts in B6 recipient mice with different treatments 14 days after transplantation. Allografts were circled with red lines. **(B)** B6 mice transplanted with a skin graft derived from BALB/c mice were treated with CsA and/or emodin for 4 weeks or until graft rejection. Graft survival was analyzed using log-rank test (*P < 0.05, *n* = 8–9 grafts).

To study the effects of emodin on allograft rejection, C57BL/6 mice received a skin graft from a donor Balb/C mouse and were then treated with emodin and/or CsA. As shown in Figure 1, we found that emodin significantly prolonged skin allograft survival compared to the control [median survival time (MST) = 24 vs. 13 days, *P* < 0.05] while allograft survival time of the recipient mice treated with CsA was also longer than that of control group (MST = 25 vs. 13 days, *P* < 0.05). More importantly, combined treatments with emodin and CsA further extended skin allograft survival compared to the treatment with either CsA or emodin alone (MST = 36 vs. 25 or 24 days, both *P* < 0.05).

**Figure 2:**
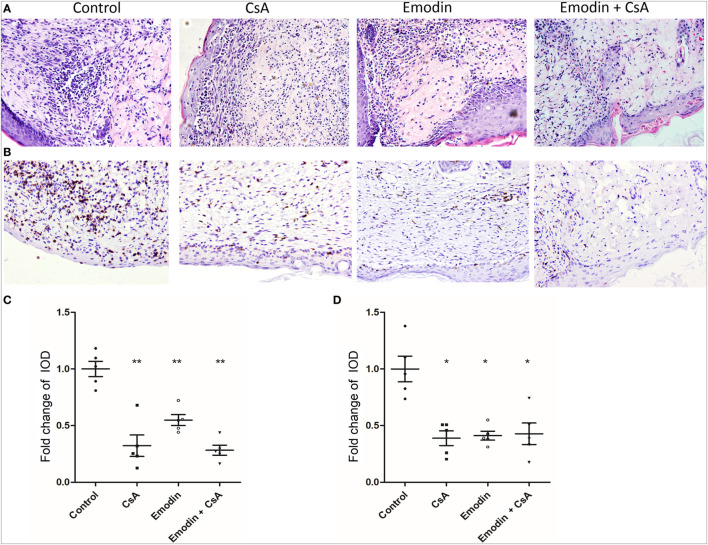
Emodin ameliorates cellular infiltration in an allograft. **(A)** H&E staining of skin allografts 10 days after transplantation (Magnification:200×). Representative images of skin graft sections are presented. **(B)** Immunohistochemistry was performed to detect infiltrating CD3^+^ T cells in skin grafts. Sections of skin grafts were stained with CD3-specfic antibody and imaged. Representative images of graft sections are presented. **(C)** The area of infiltration or inflammation in an allograft was quantified as integrated optical density (IOD) for HE-stained sections by ImagePro plus (*n* = 5 grafts). **(D)** The IOD of CD3-positive area in the images (*n* = 5 grafts) also was calculated by ImagePro plus (magnification at 200×). Data are shown as means ± SD for panels **(C,D)** (*P < 0.05, **P < 0.01).

Since emodin suppressed allograft rejection, we asked whether emodin would reduce cellular infiltration in an allograft. Graft-infiltrating cells were analyzed by H&E and immunohistochemical staining 10 days after transplantation. H&E staining showed obvious cellular infiltration in a skin allograft of the recipients without any treatment while much less cellular infiltration was observed in the recipients treated with emodin, CsA or both of them (Figures 2A,C). Similarly, immunohistochemistry revealed an obvious decrease in CD3^+^ T cell infiltration in a skin allograft of the recipients treated with emodin and/or CsA compared to that of control recipients (Figures 2B,D). These data suggest that emodin indeed suppresses allograft rejection and ameliorates alloreactive CD3^+^ T cell infiltration in the skin allografts.

### Emodin Inhibits the Expansion of Effector CD8^+^ T Cells Posttransplantation

**Figure 3:**
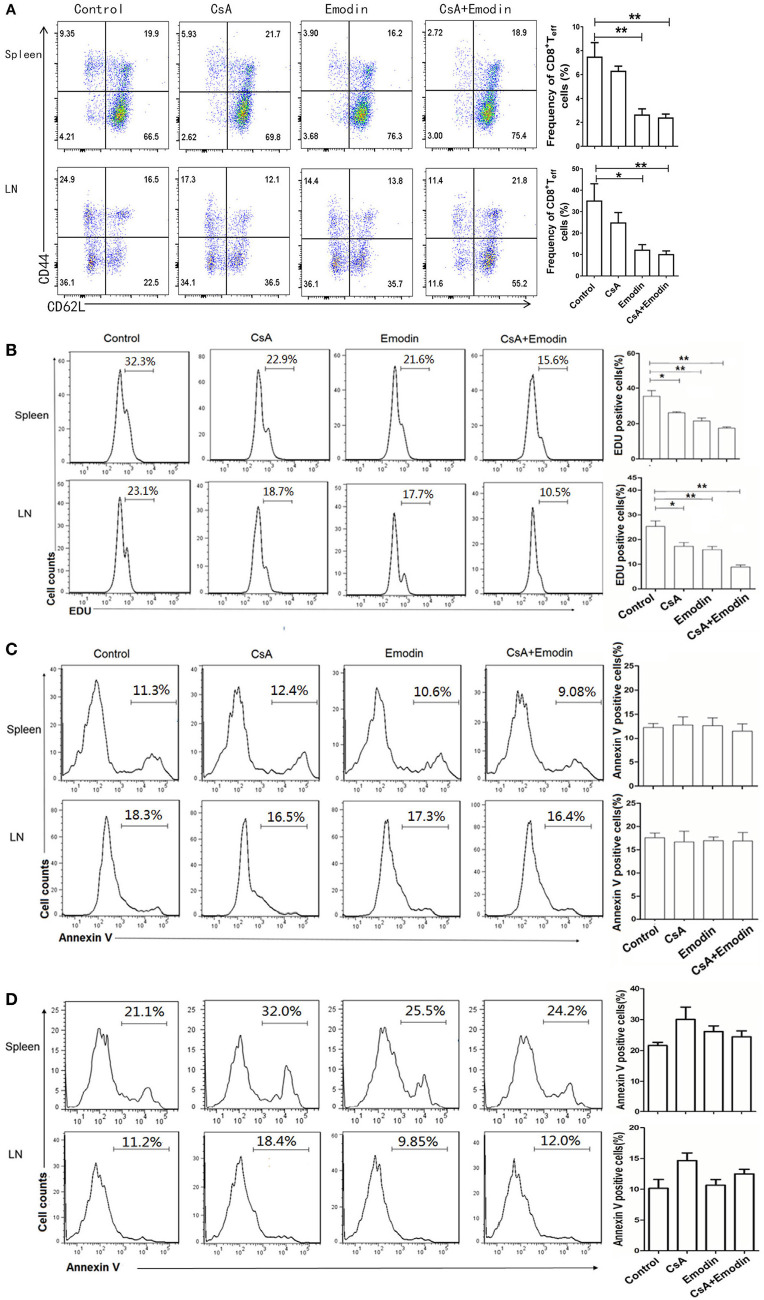
Emodin suppresses the expansion of effector CD8^+^ T cells. **(A)** Draining lymph node (LN) and spleen cells from emodin- and CsA-treated B6 mice transplanted with BALB/c skin were isolated 10 days after transplantation and analyzed *via* FACS analysis. Column graphs show the percentages of CD8^+^CD44^high^CD62L^low^ effector T cells (Teff) from LNs and spleens. **(B)** The proliferation of CD8^+^CD44^high^CD62L^low^ T cells was analyzed by EDU labeling. Recipient mice were pulsed intraperitoneally with EDU 10 days after transplantation. 24 h later, LN and spleen cells were harvested and stained for CD8, CD44, CD62L, and EDU. Histograms are gated on CD8^+^CD44^high^CD62L^low^ population. The percentage of EDU-positive CD8^+^CD44^high^CD62L^low^ cells also is shown in column graphs. The apoptosis of CD8^+^CD44^high^CD62L^low^ **(C)** and CD4^+^CD44^high^CD62L^low^ **(D)** Teff cells was measured by annexin V labeling. LN and spleen cells were stained for CD8, CD4, CD44, CD62L, and annexin V and analyzed by flow cytometry. The percentage of Annexin V-positive CD8^+^CD44^high^CD62L^low^ and CD4^+^CD44^high^CD62L^low^ Teff cells is also shown in column graphs. Data are presented as means ± SD from two separate experiments (*P < 0.05, **P < 0.01, *n* = 4 mice/group). One representative of three separate experiments is shown for all panels.

To determine whether emodin would control effector CD8^+^ T cells (Teff) *in vivo*, draining LN and spleen cells from emodin- or CsA-treated recipient mice were isolated 10 days after transplantation and analyzed *via* EDU-staining and FACS analysis. As represented by Figure 3A, both emodin and CsA obviously decreased the percentages and absolute numbers of CD8^+^CD44^high^CD62L^low^ effector T cells in both LNs and spleens of the recipients. Furthermore, we found that either emodin or CsA alone suppressed the proliferation of CD8^+^CD44^high^CD62L^low^ T cells in the recipient mice while emodin plus CsA further inhibited their proliferation compared to either emodin or CsA alone (Figure 3B). As a control, we isolated spleen cells from naïve mice without skin transplantation and observed that there was no any difference in CD8^+^CD44^high^CD62L^low^ T cell numbers between untreated and emodin-treated groups (data not shown). On the other hand, emodin did not promote the apoptosis of effector CD8^+^CD44^high^CD62L^low^ and CD4^+^CD44^high^CD62L^low^ T cells 10 days after transplantation (Figures 3C,D). We also demonstrated that emodin did not induce the apoptosis of total CD4^+^ and CD8^+^ T cells 20 days posttransplantation (Figure S1 in Supplementary Material). Thus, our data suggest that emodin hinders effector CD8^+^ T cell expansion/proliferation, but does not induce T cell apoptosis, implying that a treatment with this dose of emodin is not cytotoxic.

### Emodin Facilitates the Generation of CD4^+^Foxp3^+^ Tregs Posttransplantation

**Figure 4:**
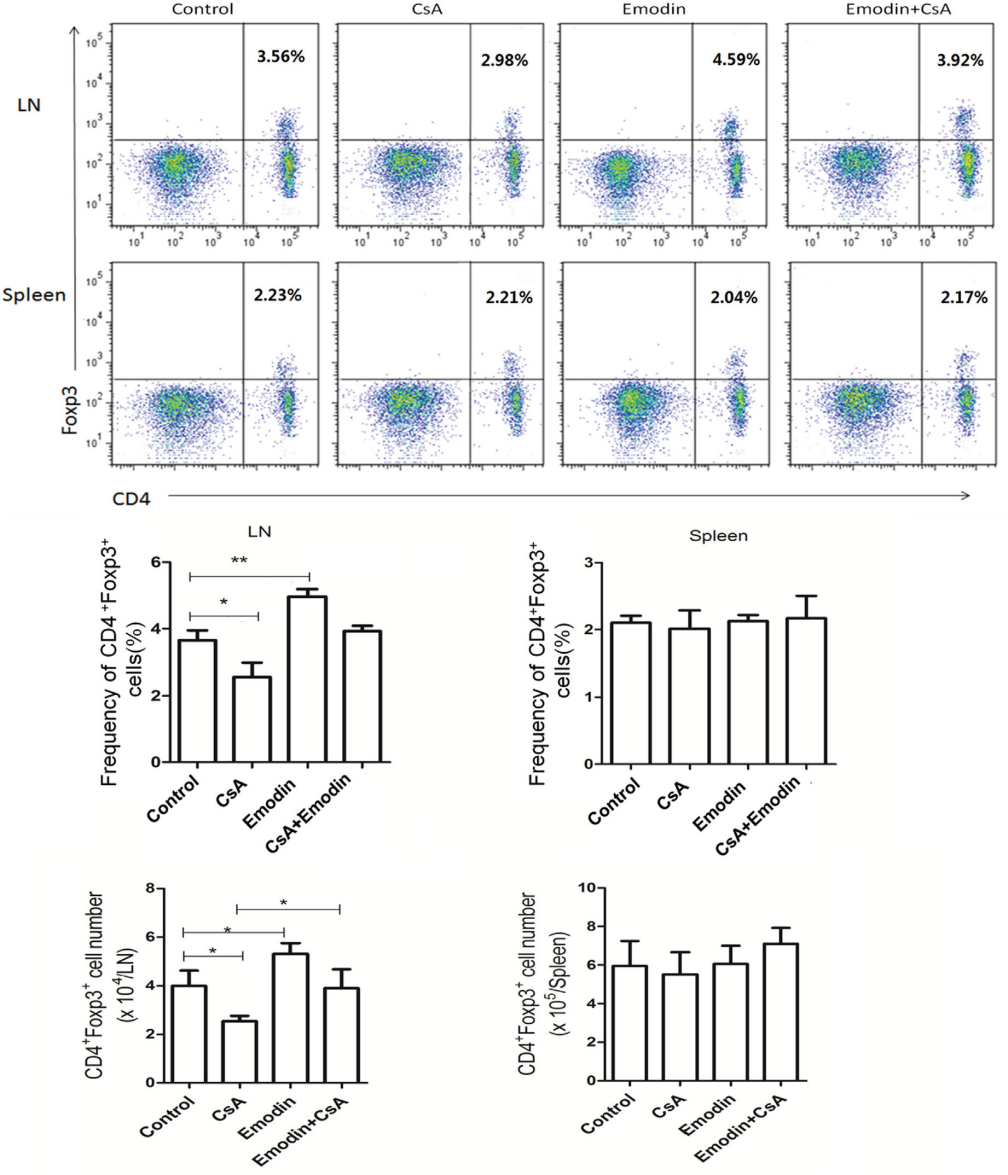
Emodin increases the frequencies and numbers of CD4^+^Foxp3^+^ regulatory T cells (Tregs). Draining lymph node (LN) and spleen cells from emodin- or CsA-treated B6 mice transplanted with BALB/c skin were isolated 10 days after transplantation. The frequency and absolute numbers of CD4^+^Foxp3^+^ Tregs from LNs and spleens of recipient mice treated with CsA and/or emodin were determined *via* a flow cytometer. Data of column graphs are shown as means ± SD (*n* = 6–8 mice). One of three separate experiments is shown.

Regulatory T cells play an important role in long-term transplant survival or tolerance. Thus, we examined if emodin would suppress allograft rejection by inducing Tregs. Draining LN and spleen cells were isolated 10 days after allogeneic skin transplantation, and CD4^+^Foxp3^+^ Tregs were enumerated by flow cytometry. As shown in Figure 4, emodin significantly increased the percentages and absolute numbers of CD4^+^Foxp3^+^ Tregs in draining LNs while CsA did the opposite. Furthermore, a reduction in LN Treg numbers resulted from CsA treatment was totally reversed in the recipients treated with both emodin and CsA (Figure 4). On the other hand, there was no markedly variance in the frequencies and absolute numbers of splenic Tregs between all groups. Similar findings were also seen 20 days after skin allotransplantation (data not shown). These data suggest that emodin promotes CD4^+^Foxp3^+^ Treg generation mainly in the draining LNs of recipient mice.

### Emodin Also Induces CD8^+^CD122^+^ Tregs

**Figure 5:**
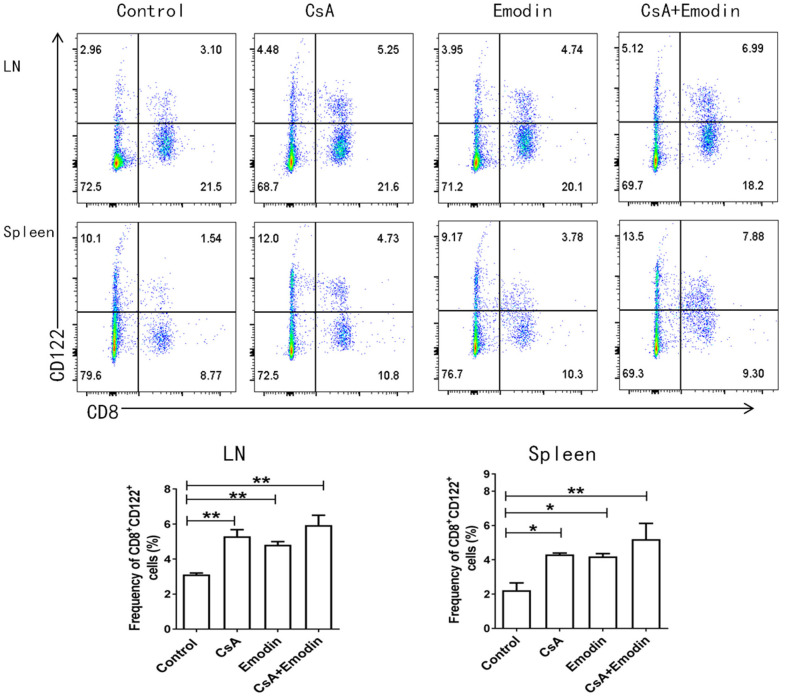
Emodin also augments the percentages of CD8^+^CD122^+^ Tregs. Draining LN and spleen cells from emodin- or CsA-treated B6 mice transplanted with BALB/c skin were isolated 10 days after transplantation. The percentages of CD8^+^CD122^+^ Tregs from LNs and spleens of recipient mice were measured *via* a flow cytometer. Data are shown as means ± SD from two separate experiments (*P < 0.05 and **P < 0.01, *n* = 4–5 mice/group).

Our previous studies demonstrated that CD8^+^CD122^+^ Tregs were more potent in inhibiting T cell proliferation and transplant rejection than CD4^+^CD25^+^ Tregs (21). Hence, we asked whether emodin would also exert its suppressive effects on allograft rejection through inducing CD8^+^CD122^+^ Tregs. We measured the percentages and absolute numbers of CD8^+^CD122^+^ Tregs in the LNs and spleens of recipient mice *via* flow cytometry 10 days after allogeneic skin transplantation. We demonstrated that both emodin and CsA significantly increased the percentages of CD8^+^CD122^+^ Tregs in both LNs and spleens of the recipients (Figure 5), suggesting that emodin generally promotes the development of CD8^+^CD122^+^ Tregs *in vivo*. Interestingly, emodin increased the absolute numbers of CD8^+^CD122^+^ Tregs in LNs, but not spleens, of the recipients.

### Emodin-Induced CD4^+^CD25^+^, but Not CD8^+^CD122^+^, Tregs Are More Potent in Suppression of Allograft Rejection than Are Control Tregs

**Figure 6:**
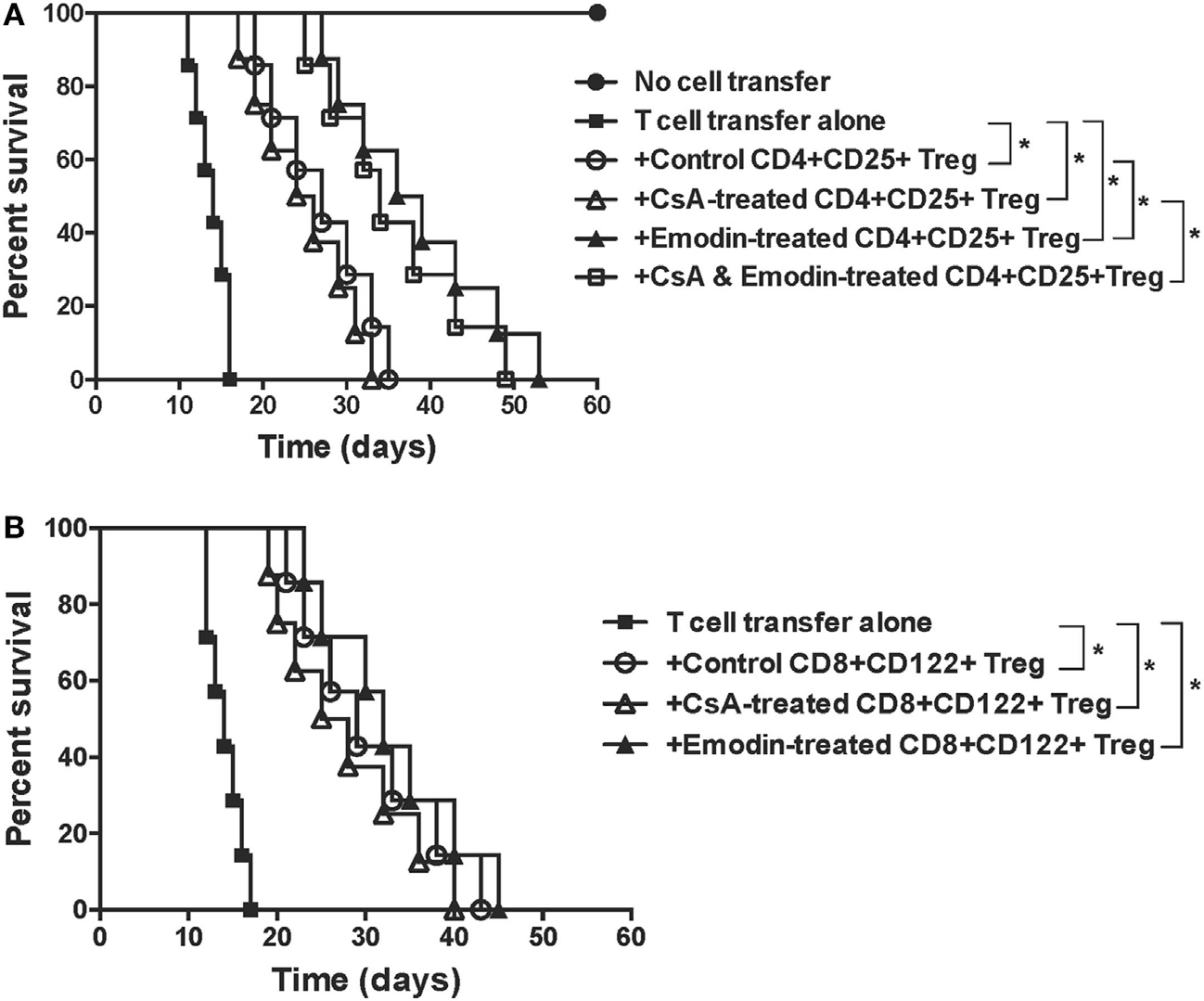
Emodin-induced CD4^+^CD25^+^ or CD8^+^CD122^+^ regulatory T cells (Tregs) suppress skin allograft rejection in Rag1^−/−^ recipient mice. CD4^+^CD25^+^ or CD8^+^CD122^+^ Tregs were isolated *via* FACS cell sorting from B6 recipient mice that were transplanted with BALB/c skin and treated with emodin and/or CsA. CD4^+^CD25^+^ **(A)** or CD8^+^CD122^+^ **(B)** Tregs (0.4 × 10^6^), together with B6-derived naïve CD3^+^ T cells (2 × 10^6^), were adoptively transferred to Rag1^−/−^ mice (B6 background) that were then transplanted with a BALB/c skin graft. Skin allograft rejection in Rag1^−/−^ recipient mice was observed (*n* = 7–8 grafts).

To determine whether emodin also enhances the suppressive function of Tregs, CD4^+^CD25^+^ or CD8^+^CD122^+^ Tregs were isolated from recipient mice treated without or with emodin and/or CsA. These Tregs were then transferred to lymphocyte-deficient Rag1^−/−^ mice that received syngeneic T cells as well as a skin allograft. As shown in Figure 6A, adoptive transfer of conventional T cells to Rag1^−/−^ recipients caused their rejection of skin allografts while control Rag1^−/−^ recipients did not reject the allografts. Transfer of both control CD4^+^CD25^+^ Tregs and T cells extended allograft survival compared to that of T cells alone. However, transfer of CD4^+^CD25^+^ Tregs derived from emodin-treated, but not CsA-treated, recipient mice resulted in longer allograft survival than that of control CD4^+^CD25^+^ Tregs derived from control recipients, suggesting that emodin enhances the suppressive function of CD4^+^CD25^+^ Tregs. Transfer of CD4^+^CD25^+^ Tregs derived from CsA- and emodin-treated recipients also caused longer allograft survival than that of the Tregs from the recipients treated with CsA alone. On the other hand, CD8^+^CD122^+^ Tregs derived from the recipient mice treated with either emodin or CsA prolonged allograft survival, but only as effectively as control CD8^+^CD122^+^ Tregs derived from control recipients (Figure 6B), indicating that emodin or CsA does not alter the suppressive capacity of CD8^+^CD122^+^ Tregs.

### Depletion of Tregs Largely Reverses Allograft Survival Extended by Emodin

**Figure 7:**
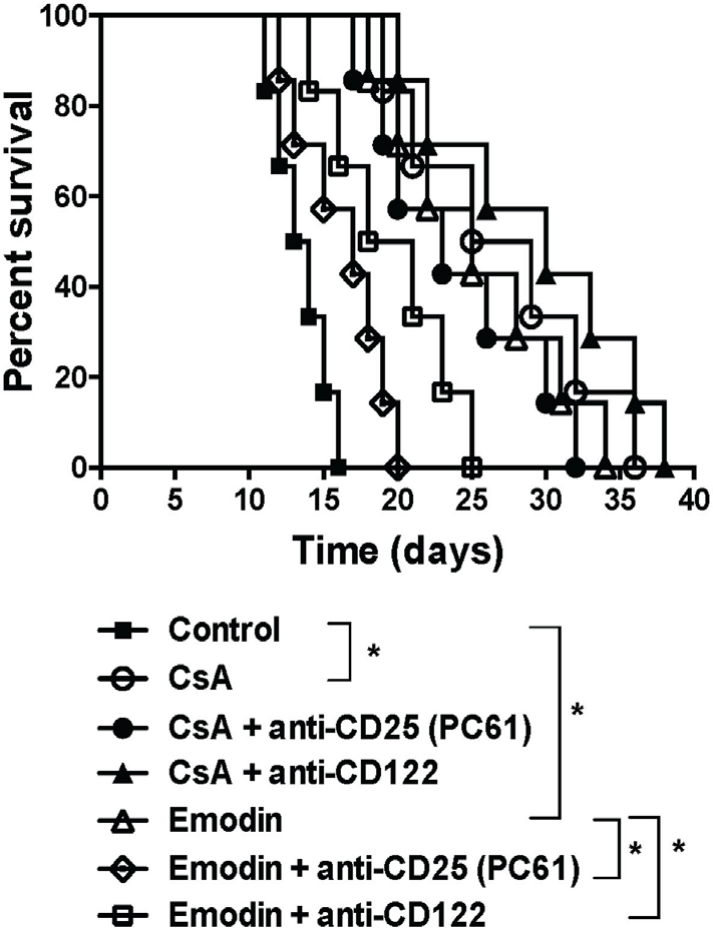
Depleting regulatory T cells (Tregs) largely reverses allograft survival extended by emodin. C57BL/6 mice received a skin graft from a BALB/c mouse and were treated with emodin or CsA. Tregs in recipient mice were depleted using anti-CD25 (PC61) and anti-CD122 (TMβ1). Skin allograft rejection was then observed (*n* = 6–7 grafts).

Given that emodin induced Tregs in our animal model, we asked whether the effects of emodin on allograft survival were dependent on the Tregs. C57BL/6 mice received a skin graft from a BALB/c mouse and were treated with emodin or CsA. Tregs in the recipients then were depleted using anti-CD25 (PC61) or anti-CD122 (TMβ1) Ab. As shown in Figure 7, depleting CD25^+^ Tregs largely reversed skin allograft survival prolonged by emodin while depleting CD122^+^ Tregs partially abrogated the allograft survival extended by emodin. In contrast, depletion of either CD25^+^ or CD122^+^ Tregs did not significantly shorten allograft survival induced by CsA. As control experiments, isotype control Abs did not alter allograft survival (data not shown). Our results suggest that Tregs contributes to allograft survival induced by emodin.

### Emodin Suppresses Alloantibody Production Posttransplantation

**Figure 8:**
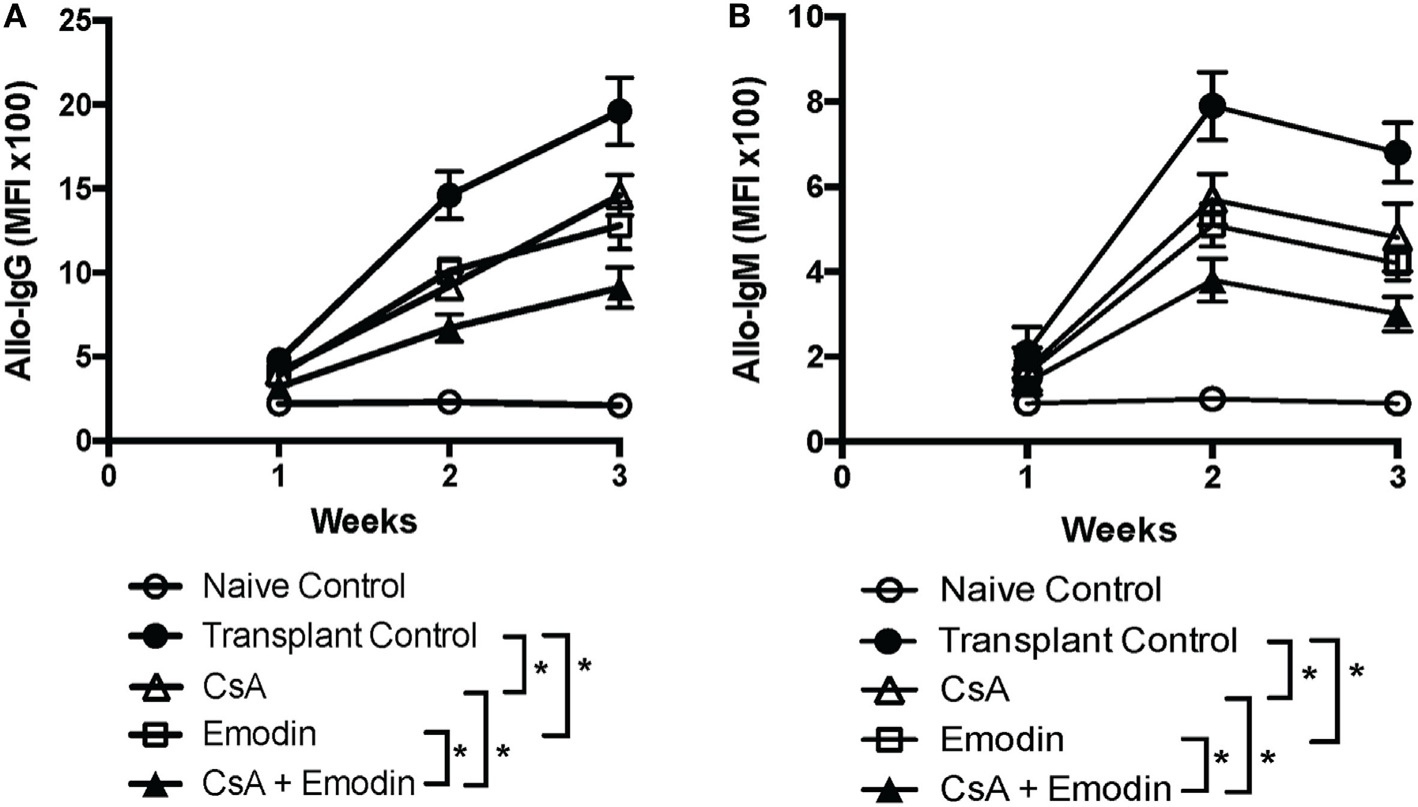
Emodin suppresses alloantibody production posttransplantation. IgG **(A)** and IgM **(B)** in the serum of B6 recipient mice treated with CsA and/or emodin were measured *via* FACS analysis using B-cell-depleted BALB/c splenocytes as primary target cells 1–3 weeks posttransplantation. The data were pooled from three separate experiments and presented as means ± SD (*n* = 6–8 mice) in the form of the mean fluorescence intensity (MFI).

Given that anti-donor antibodies play a role in allograft rejection, we examined whether emodin also reduced donor-specific antibodies. We then measured IgG and IgM in the serum of B6-recipient mice treated with CsA and/or emodin *via* FACS analyses using BALB/c splenocytes as target cells. As shown in Figure 8, either emodin or CsA significantly lowered both IgG and IgM levels 2 and 3 weeks after transplantation while administration of both CsA and emodin further decreased IgG and IgM levels compared to the treatment with CsA or emodin alone. Our data indicate that emodin indeed inhibits alloantibody production.

### Emodin Hinders DC Maturation Posttransplantation

**Figure 9:**
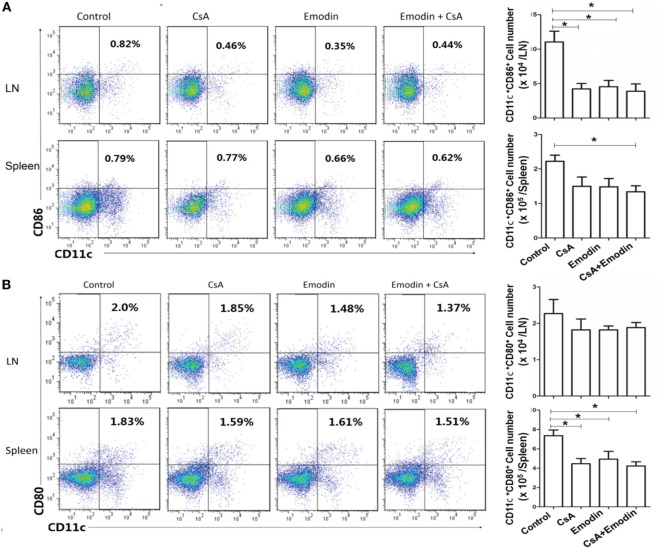
Emodin hinders dendritic cell (DC) maturation posttransplantation. Draining lymph node (LN) and spleen cells were isolated from B6 recipient mice that were transplanted with BALB/c skin and treated with CsA and/or emodin. 10 days after allogeneic skin transplantation, CD11c^+^CD86^+^ **(A)** and CD11c^+^CD80^+^ **(B)** DCs in recipients were enumerated by flow cytometry. Bar graphs represent the absolute numbers of the DCs. Data were pooled from three separate experiments and presented as means ± SD (*n* = 6–9 mice).

To determine if emodin affects DC maturation posttransplantation, draining LN and spleen cells were isolated 10 days after allogeneic skin transplantation, and CD11c^+^CD80^+^ and CD11c^+^CD86^+^ DCs were enumerated by flow cytometry. As shown in Figure 9, treatment with either CsA or emodin reduced CD11c^+^CD86^+^ cell numbers in LNs of the recipient mice. However, combined treatments with both CsA and emodin, but not treatment with CsA or emodin alone, significantly decreased CD11c^+^CD86^+^ cell numbers in spleens of the mice (Figure 9A). On the other hand, either CsA or emodin reduced CD11c^+^CD80^+^ cell numbers in spleens, but not LNs, of the recipients (Figure 9B). These findings suggest that emodin reduces CD11c^+^CD86^+^ DC numbers in LNs of recipient mice while decreasing CD11c^+^CD80^+^ DC numbers in spleens of the recipients.

### Emodin Suppresses T Cell Proliferation and mTOR Signaling *In Vitro*

**Figure 10:**
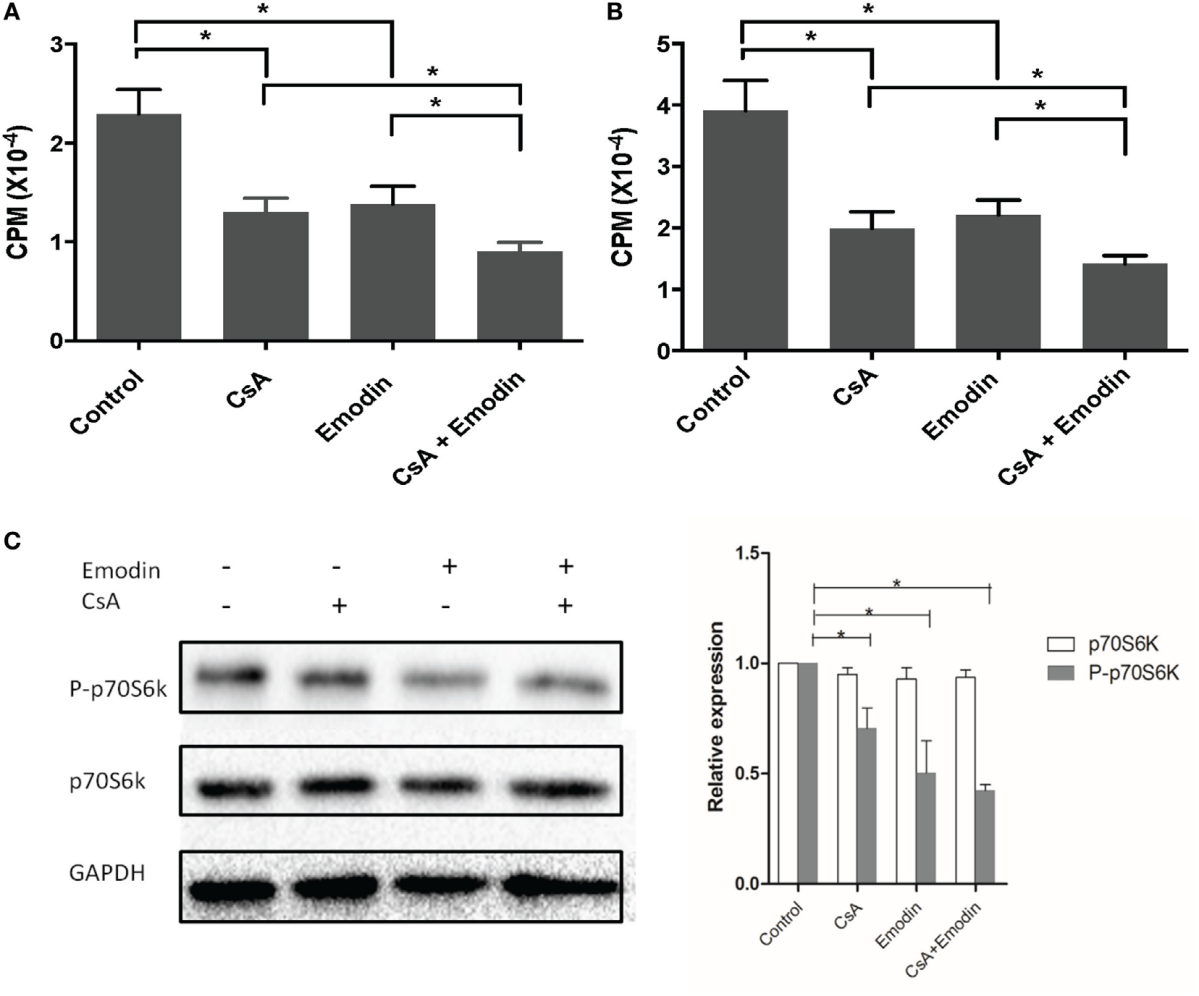
Emodin inhibits T cell proliferation and mTOR signaling *in vitro*. An MLR was set up using irradiated Balb/C splenocytes as stimulators and B6-derived T cells as responders in the absence or presence of CsA and/or or emodin. Either emodin or CsA inhibited T cell proliferation 3 **(A)** or 5 days **(B)** after MLR culture. Data are shown as means ± SD (*n* = 6 samples). One representative of three separate experiments is shown. Moreover, western blots for phosphorylated p70S6K and total p70S6K were performed two days after the similar MLR culture **(C)**. GAPDH was used as a loading control. One representing image from three separate experiments is shown. OD values in column graphs were pooled from four separate experiments. Data are presented as means ± SD (*n* = 4 bands) after corrections by GAPDH. Results demonstrated that emodin mostly blocked the phosphorylation of p70S6K.

Since we found that emodin suppressed allograft rejection and T cell infiltration in an allograft, we then examined whether emodin would also inhibit T cell proliferation *in vitro*. To determine the effects of emodin on alloreactive T cell proliferation *in vitro*, one-way MLR was set up using irradiated Balb/C splenocytes as stimulators and B6-derived T cells as responders. As shown in Figure 10, either emodin or CsA inhibited T cell proliferation 3 (Figure 10A) or 5 days (Figure 10B) after MLR culture. Moreover, combined treatments with both emodin and CsA further suppressed T cell proliferation compared to the treatment with emodin or CsA alone.

Previous experiments using rapamycin, an inhibitor of mTOR (22), have proved that mTOR signal transduction suppresses the development and function of Tregs and that rapamycin promotes CD4^+^Foxp3^+^ Treg generation (23). Since our results demonstrated that emodin increased the frequencies of CD4^+^Foxp3^+^ Tregs, we determined whether emodin would also induce Tregs by blocking mTOR signaling. T cells were activated in an MLR in the medium without or with emodin for 48 h, and the phosphorylation of p70S6K protein (P-p70S6K) was measured *via* western blotting. As shown in Figure 10C, emodin effectively inhibited the phosphorylation of downstream protein p70S6K at Ser411 compared to the control group while CsA only slightly reduced its phosphorylation. These results indicate that emodin likely exerts its effects on Treg generation and T cell proliferation through blocking mTOR signaling pathway.

## Discussion

CsA has been widely used as an immunosuppressive agent in clinic for treating both autoimmune diseases and transplant rejection. However, in addition to its common side effects, CsA hinders allograft tolerance by dampening CD4^+^CD25^+^FoxP3^+^ Tregs (24, 25). Therefore, it is necessary to develop new therapeutic drugs for suppressing allograft rejection with few side effects. In particular, an immunosuppressive agent that spares Tregs is highly preferred. Using a mouse model of skin transplantation, we analyzed the efficacy of emodin, a natural molecule extracted from *R. palmatum*, in suppression of transplant rejection. Our data demonstrated that emodin suppressed T cell infiltration in an allograft and prolonged the allograft survival. Further, administration of emodin induced both CD4^+^FoxP3^+^ and CD8^+^CD122^+^ Tregs while inhibiting the expansion of effector CD8^+^ T cells in transplanted mice. Emodin also suppressed alloantibody production and hindered DC maturation posttransplantation. Finally, emodin suppressed T cell proliferation and mTOR signaling *in vitro*. Thus, emodin may represent a newly emerging immunosuppressant that could be utilized in clinical transplantation.

Our results demonstrated that emodin suppressed effector CD8^+^ T cell expansion but did not promote CD4^+^ and CD8^+^ T cell apoptosis in recipient mice, suggesting that emodin is non-cytotoxic in our animal model. However, a previous study showed that emodin induced the apoptosis of Dalton’s lymphoma cells *in vivo via* modulating hydrogen peroxide metabolizing antioxidant enzymes (26). Although it is unclear why emodin induced the apoptosis of lymphoma cells, but not primary T cells, we speculate that rapidly growing tumor cells may be more vulnerable to mitochondrial pathway of apoptosis than normal primary T cells in wild-type mice.

CD4^+^CD25^+^ Tregs play a key role in maintaining immune homeostasis and tolerance. They represent only a small fraction of CD4^+^ T cells and express α chain of IL-2 receptor (CD25) (27–29). Either induction of endogenous CD4^+^CD25^+^ Tregs or adoptive transfer of exogenous Tregs prevents autoimmune diseases and allograft rejection in animal models (30–37). Here, we found that emodin upregulated CD4^+^Foxp3^+^ Tregs in the draining LNs of transplanted mice while CsA reduced their numbers, suggesting that emodin and CsA suppress allograft rejection *via* a totally different mechanism. CsA is a common immunosuppressant for suppression of allograft rejection through the formation of a cyclosporin–cyclophilin complex and prevention of T cell activation (38), resulting in reduced IL-2 expression, impaired generation of CD4^+^Foxp3^+^ Tregs (39, 40) and compromised tolerance. In our studies, CsA treatment led to similar reductions in the frequencies and numbers of Tregs, which were reversed by emodin administration, suggesting that emodin can correct the deficiency of CsA in terms of its repression of Tregs. Thus, potential tolerance-breaking effects of CsA may be offset by emodin. It is unclear why the frequencies and numbers of these Tregs in spleens were not affected by emodin. It is possible that an increase in Tregs in the draining LNs of the recipient mice treated with emodin was due to the presence of alloantigen-specific Tregs given that Ag-specific T/Treg cell frequencies are generally lower in spleens than in the draining LNs. On the other hand, depletion of CD4^+^Foxp3^+^ Tregs mostly reversed allograft survival extended by emodin, indicating that suppression of allograft rejection by emodin is mostly dependent on CD4^+^Foxp3^+^ Tregs.

Recent studies have shown that CD8^+^CD122^+^ T cells are also Tregs that suppress conventional T cell responses (41–46) and autoimmune diseases (47, 48). We have previously demonstrated that CD8^+^CD122^+^ T cells not only are Tregs (49, 50), but also are more potent in suppression of allograft rejection than conventional CD4^+^CD25^+^ Tregs (21). In this study, we found that both emodin and CsA significantly increased the frequency of CD8^+^CD122^+^ Tregs in both LNs and spleens of the recipients, indicating that the signaling pathways activating CD8^+^CD122^+^ Tregs are different from those activating CD4^+^Foxp3^+^ Tregs. Interestingly, emodin augmented the absolute number of CD8^+^CD122^+^ Tregs in the LNs, but not the spleen. It is possible that most Tregs induced by emodin migrated to the draining LNs or the allograft. On the other hand, neither emodin nor CsA enhanced the suppressive capacity of CD8^+^CD122^+^ Tregs, implying that emodin-induced CD8^+^CD122^+^ Tregs are not necessarily alloantigen-specific. Furthermore, depletion of CD8^+^CD122^+^ Tregs only partially reversed allograft survival induced by emodin, indicating that inhibition of allograft rejection by emodin is not totally dependent on CD8^+^CD122^+^ Tregs.

We demonstrated that emodin induced CD4^+^Foxp3^+^ Tregs while inhibiting conventional T cell proliferation. It has been well accepted that mTOR signaling is important for regulating the generation of CD4^+^Foxp3^+^ Tregs and that an mTOR inhibitor, such as rapamycin, induces Tregs (7, 8). We, therefore, speculated that emodin exerted its suppressive effects on T cell responses *via* inhibition of mTOR signaling, leading to an increase in the Treg generation. Indeed, we found that emodin-treated T cells exhibited a dramatic decrease in the activation of their mTOR signaling, which is induced by TCR stimulation (51), and also a reduction in their proliferation, indicating that emodin suppresses allograft rejection by blocking T-cell mTOR signaling, leading to the Treg generation, which in turn prolongs allograft survival.

Donor-specific antibodies or alloantibodies play an important role in mediating allograft rejection. We found that emodin suppressed the production of both IgG and IgM alloantibodies after allotransplantation. The mechanisms underlying its suppression of alloantibody production are unknown. It is possible that T cell help for B cell differentiation and function is compromised due to the suppression of T cell activation by emodin.

Dendritic cells play an important role in T cell activation and CD11c-expressing DCs in recipients facilitate allograft rejection and promote the expansion of alloreactive CD4^+^ and CD8^+^ T cells (52). In our studies, we found that emodin reduced CD11c^+^CD86^+^ DC numbers in LNs of recipient mice while decreasing CD11c^+^CD80^+^ DC numbers in spleens of the recipients, suggesting that emodin hinders DC maturation in the context of allotransplantation. It remains to be determined why emodin differentially impacts CD11c^+^CD86^+^ and CD11c^+^CD80^+^ subsets of DCs in the LNs and spleens of the recipient mice. However, our results are actually consistent with a previous study showing that emodin inhibited DC maturation *in vitro* (53), implying that emodin indeed suppresses DC differentiation or maturation. It is also unclear how emodin reduces DC numbers. It is possible that emodin regulates the expression of various toll-like receptors.

In conclusion, emodin inhibits alloimmune responses by inducing Tregs, suppressing alloantibody production, hindering DC maturation, and blocking mTOR signaling. It remains to be defined if emodin also regulates the generation and function of other innate immune cells, including MDSCs, macrophages, and NK cells. Our results will lay the groundwork for clinical trials using emodin as an effective immunosuppressant to suppress allograft rejection or even autoimmunity.

## Ethics Statement

This study was carried out in accordance with the recommendations of the “Chinese national guidelines for the Care and Use of Laboratory Animals” and the protocol was approved by “the Institutional Animal Care and Use Committee of Guangdong Provincial Academy of Chinese Medical Sciences.”

## Author Contributions

FQ designed and performed experiments; HL and C-LL conducted partial experiments; GN provided idea and edited the manuscript; and ZD designed experiments and wrote the paper.

## Conflict of Interest Statement

The authors declare that the research was conducted in the absence of any commercial or financial relationships that could be construed as a potential conflict of interest.
