# Corrigendum: A New Immunosuppressive Molecule Emodin Induces both CD4^+^FoxP3^+^ and CD8^+^CD122^+^ Regulatory T Cells and Suppresses Murine Allograft Rejection

**DOI:** 10.3389/fimmu.2020.01381

**Published:** 2020-07-16

**Authors:** Feifei Qiu, Huazhen Liu, Chun-Ling Liang, Golay D. Nie, Zhenhua Dai

**Affiliations:** ^1^Section of Immunology, Guangdong Provincial Academy of Chinese Medical Sciences, Guangdong Provincial Hospital of Chinese Medicine, Guangzhou, China; ^2^School of Medicine, University of Texas Medical Branch, Galveston, TX, United States

**Keywords:** transplant immunology, regulatory T cell, T cell, immunosuppression, allograft rejection

In the original article, there were errors in Figure 3A and Figure 5 as published. As such, minor adjustments were made with **“*n* = 4-5 mice”** now, which is reflected in the legends below. In addition, there were irregular clusters of dots between the two adjacent groups of LN in Figure 3A and Spleen in Figure 5. We decided to abandon this set of inaccurate data while using two additional sets of the raw data that do not have any problem. We therefore provided the representing dot plots from one of the two sets of the correct raw data for the corrections and also updated the column graphs accordingly. The corrected figures and legends appear below.

**Figure 3A:**
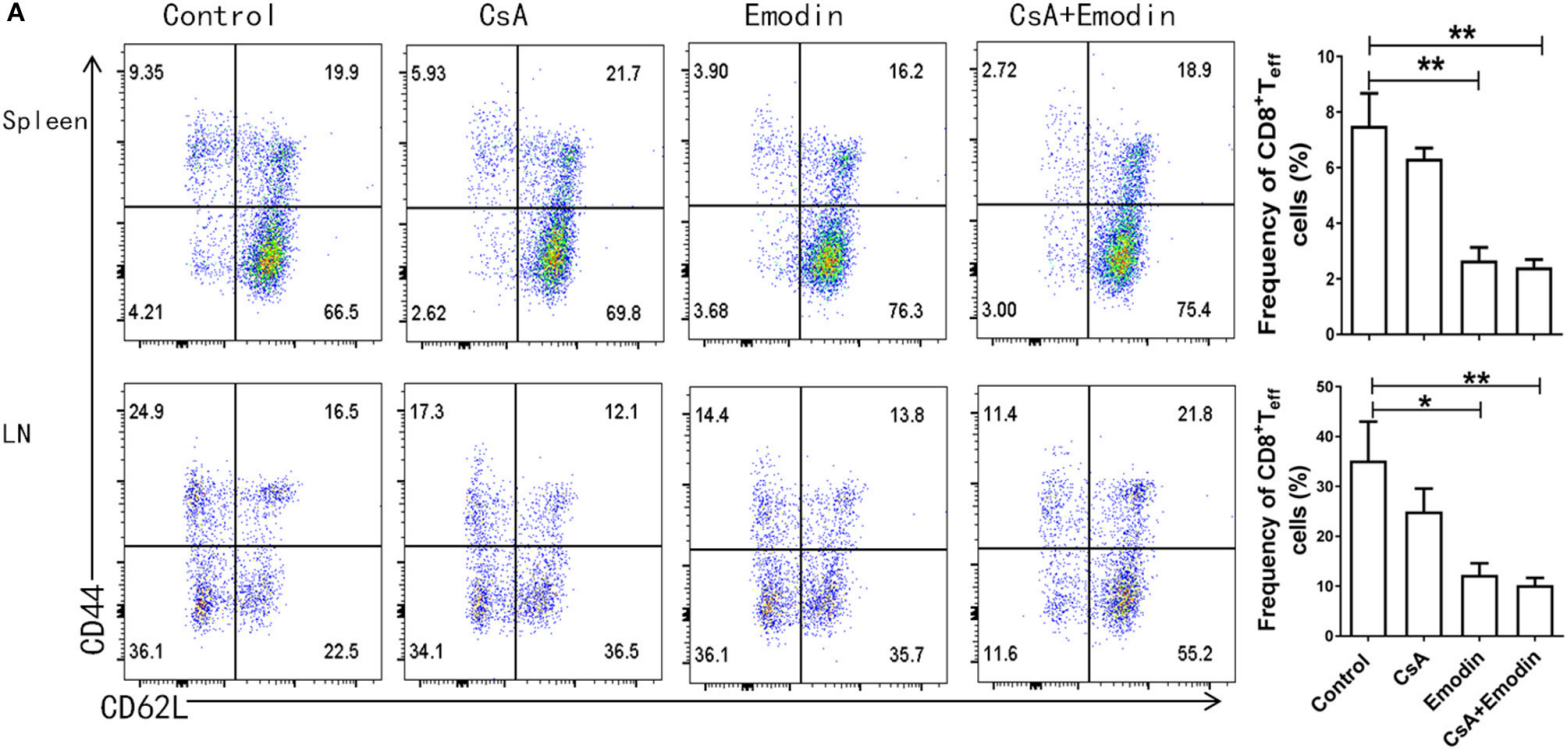
Emodin suppresses the expansion of effector CD8^+^ T cells. **(A)** Draining lymph node (LN) and spleen cells from emodin- and CsA-treated B6 mice transplanted with BALB/c skin were isolated 10 days after transplantation and analyzed *via* FACS analysis. Column graphs show the percentages of CD8^+^CD44^high^CD62L^low^ effector T cells (Teff) from LNs and spleens. Data are presented as means ± SD from two separate experiments (*P < 0.05, **P < 0.01, *n* = 4 mice/group).

**Figure 5:**
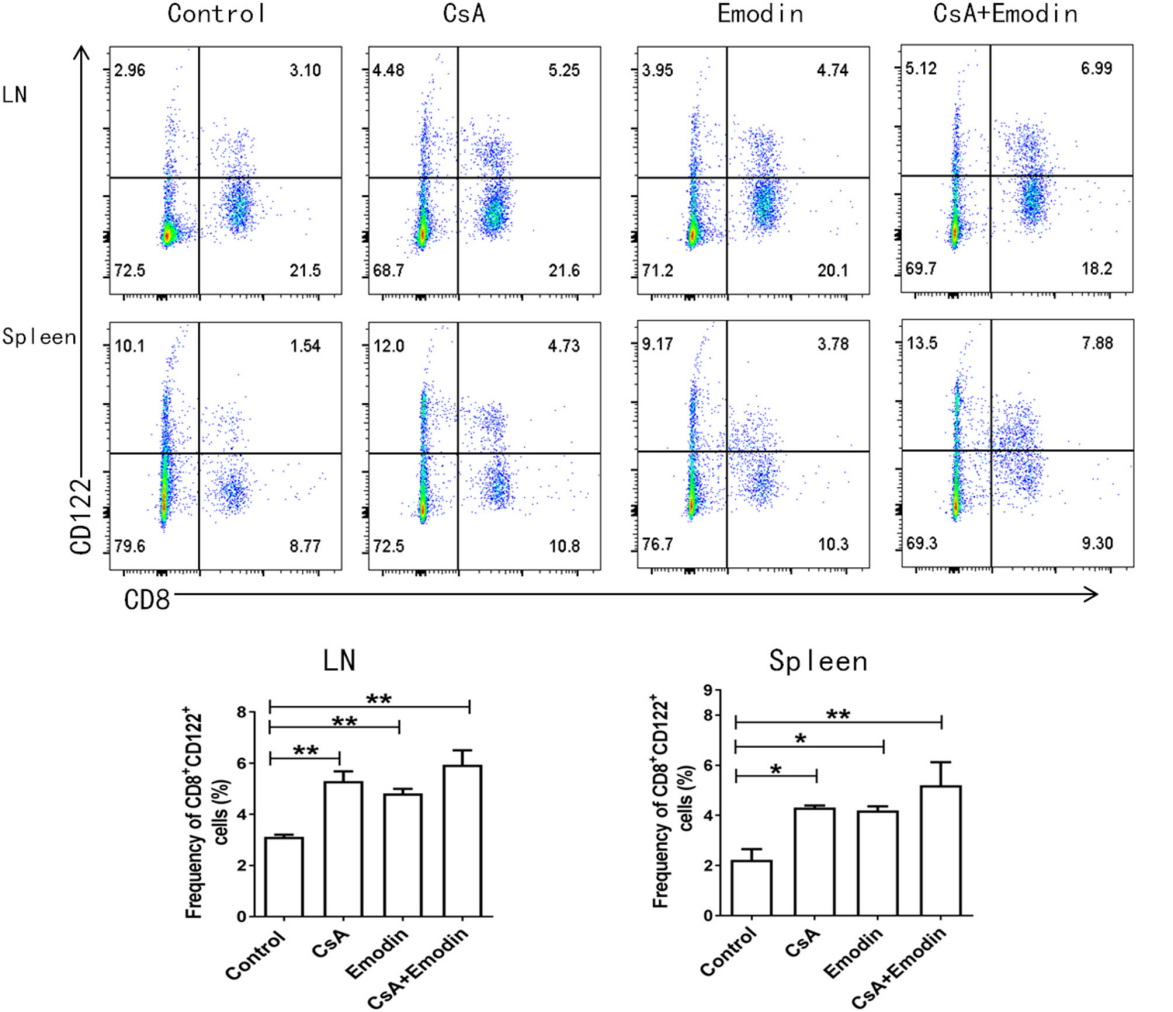
Emodin also augments the percentages of CD8^+^CD122^+^ Tregs. Draining LN and spleen cells from emodin- or CsA-treated B6 mice transplanted with BALB/c skin were isolated 10 days after transplantation. The percentages of CD8^+^CD122^+^ Tregs from LNs and spleens of recipient mice were measured *via* a flow cytometer. Data are shown as means ± SD from two separate experiments (*P < 0.05 and **P < 0.01, *n* = 4–5 mice/group).

The authors apologize for this error and state that this does not change the scientific conclusions of the article in any way. The original article has been updated.

