# Supplementary material for: A New Immunosuppressive Molecule Emodin Induces both CD4+FoxP3+ and CD8+CD122+ Regulatory T Cells and Suppresses Murine Allograft Rejection
Source: Front Immunol. 2017 Nov 8;8:1519. doi: 10.3389/fimmu.2017.01519 (PMC5682309; doi:10.3389/fimmu.2017.01519)
Supplement: Supplementary file 1 [file Presentation_1.pdf]

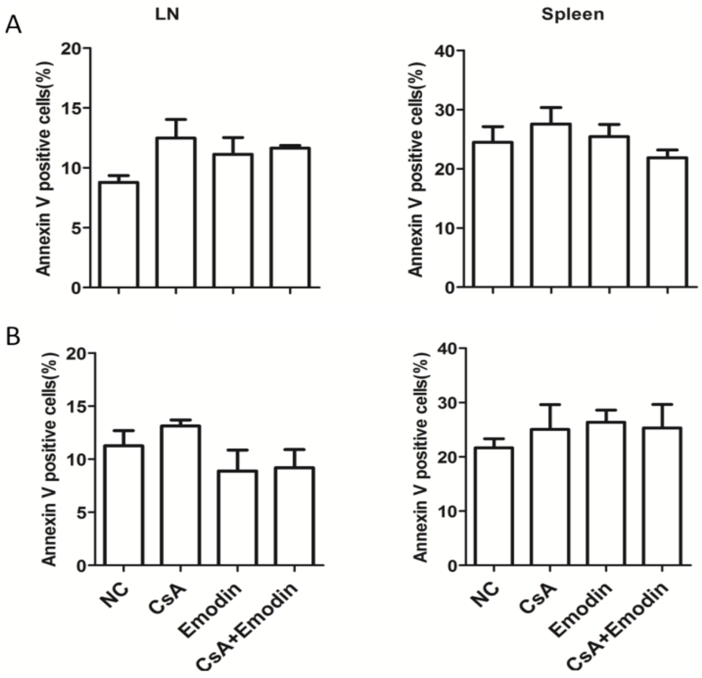

Supplementary Figure 1

Emodin does not induce the apoptosis of $CD4^+$ and $CD8^+$ T cells in vivo

The apoptosis of $CD4^+$ (**A**) and $CD8^+$ (**B**) T cells in recipient mice was measured by Annexin V labeling 20 days after skin transplantation and treatments with CsA and/or emodin. LN and spleen cells were harvested and stained for CD4, CD8 and annexin V and their apoptosis was analyzed by flow cytometry. The percentages of Annexin V-positive $CD4^+$ and $CD8^+$ T cells are shown. Data are presented as means ± S.D (n=5-6). Results were pooled from three separate experiments. Our findings suggest that administration of emodin does not promote $CD4^+$ and $CD8^+$ T cell apoptosis in recipient mice.
